# Supplementary material for: Proteomic Response of Paracoccidioides brasiliensis Exposed to the Antifungal 4-Methoxynaphthalene-N-acylhydrazone Reveals Alteration in Metabolism
Source: J Fungi (Basel). 2022 Dec 31;9(1):66. doi: 10.3390/jof9010066 (PMC9865261; doi:10.3390/jof9010066)
Supplement: Supplementary file 1 [file jof-09-00066-s001.zip › jof-2089857-supplementary.pdf]

Supplementary material

Changes in proteomic profile of *Paracoccidioides brasiliensis* induced by 4-methoxynaphthalene-*N*-acylhydrazone

Lívia do Carmo Silva[1*], Kleber Santiago Freitas e Silva[1*], Olívia Basso Rocha[*], Katheryne Lohany Barros Barbosa[1], Andrew Matheus Frederico Rozada[2], Gisele de Freitas Gauze[2], Célia Maria de Almeida Soares[1], Maristela Pereira[1**]

[1] Laboratory of Molecular Biology, Institute of Biological Sciences, Federal University of Goiás, Goiânia, Goiás, Brazil.
[2] Department of Chemistry, State University of Maringa, Maringá, Paraná, Brazil
*Contributed equally to this work
**Correspondent author: Lívia do Carmo Silva – liviacarmo.bio@gmail.com, Maristela Pereira - maristelaufg@gmail.com, Kleber Santiago Freitas e Silva – smallbinho@hotmail.com, Laboratory for Molecular Biology, Federal University of Goiás, Avenida Esperança, s/n, ICB2, Sala 206, Goiânia, GO, 74690-900, Brazil; Telephone: +556235211110.

Table S1. Proteins upregulated by *P. brasiliensis* after exposure to AOS.

Access Number	Biological process	Fold change
**METABOLISM**		
**Metabolism aminoacid**		
PADG_05085	1-pyrroline-5-carboxylate dehydrogenase	1.36
PADG_02914	Aminomethyltransferase	1.46
PADG_06546	Aminopeptidase	T
PADG_00637	Arginase	1.40
PADG_04167	Aspartyl aminopeptidase	T
PADG_04142	Acylpyruvate hydrolase	T
PADG_01418	Cysteine dioxygenase	T
PADG_01479	Gamma-glutamyltransferase	T
PADG_04516	Glutamate dehydrogenase	T
PADG_05337	Glutamate-5-semialdehyde dehydrogenase	T
PADG_00663	Homoserine dehydrogenase	T
PADG_07031	Hydroxymethylglutaryl-CoA lyase	T
PADG_01328	Ornithine aminotransferase	T
PADG_05820	Probable Xaa-Pro aminopeptidase P	T
PADG_01314	Pyridoxal phosphate homeostasis protein	T
PADG_01718	Saccharopine dehydrogenase [NADP+. L-glutamate-forming]	T
PADG_08406	O-acetylhomoserine (Thiol)-lyase	1.32
**Lipid, fatty acid and isoprenoid metabolism**		
PADG_08018	Glycerol-3-phosphate dehydrogenase	T
PADG_04495	4-coumarate-CoA ligase	T
PADG_03194	3-ketoacyl-CoA thiolase B	T
PADG_06382	Acetyl-CoA acetyltransferase	1.35
PADG_07023	Carnitine O-acetyltransferase	T

**Nitrogen, sulfur and selenium metabolism**		
PADG_00331	Uricase	T
PADG_03871	Urease	T
PADG_06490	Formamidase	T
**Nucleotide Metabolism**		
PADG_05643	Ribonucleoside-diphosphate reductase small chain	T
PADG_00144	U6 snRNA-associated Sm-like protein LSm2	T
PADG_01100	Uracil phosphoribosyltransferase	T
PADG_07530	Thymidylate kinase	T
PADG_07782	Deoxyuridine 5'-triphosphate nucleotidohydrolase	2.29
**Metabolism of vitamins, cofactors, and prosthetic groups**		
PADG_03983	6.7-dimethyl-8-ribityllumazine synthase	1.34
PADG_02513	Fe-S protein assembly co-chaperone HscB	2.36
**ENERGY**		
**Glycolysis and gluconeogenesis**		
PADG_03813	Hexokinase	T
PADG_00192	6-phosphofructokinase	T
PADG_01278	Pyruvate kinase	1.49
PADG_02063	Pyruvate dehydrogenase	1.46
PADG_08503	Phosphoenolpyruvate carboxykinase [ATP]	T
**Tricarboxylic-acid pathway**		
PADG_04710	Citrate synthase	1.31
PADG_04249	Isocitrate dehydrogenase [NADP]	1.60
PADG_01762	Oxoglutarate dehydrogenase E1 component	T
**Electron transport and membrane-associated energy conservation**		
PADG_03872	NADH-cytochrome b5 reductase	T
PADG_11981	V-type proton ATPase catalytic subunit A	T
**Fermentation**		
PADG_00171	L-lactate dehydrogenase	T
PADG_11405	Alcohol dehydrogenase 1	T
**Pentose phosphate pathway**		
PADG_07771	6-phosphogluconolactonase	T
PADG_07420	Transaldolase	1.31
**Glyoxylate cycle**		
PADG_04702	Malate synthase	T
**BIOGENESIS OF CELLULAR COMPONENTS**		
PADG_00604	Phosphoacetylglucosamine mutase	T
PADG_04148	Alpha-mannosidase	T
PADG_04761	Mannosyl-oligosaccharide glucosidase	T
PADG_07249	Coronin	1.45
**CELL RESCUE. DEFENSE AND VIRULENCE**		
PADG_03276	S-(hydroxymethyl)glutathione dehydrogenase	T

PADG_01551	Thioredoxin reductase	1.31
PADG_01954	Superoxide dismutase	T
PADG_03423	Glutathione S-transferase Gst3	T
PADG_03562	Chaperone DnaK	1.35
PADG_07460	Vacuolar aminopeptidase	T
**CELL CYCLE**		
PADG_11679	Proliferating cell nuclear antigen	1.38
PADG_02300	Phosphatase 2C Ptc3	T
PADG_00656	Non-histone chromosomal protein 6	5.26
**TRANSCRIPTION**		
PADG_06856	Small nuclear ribonucleoprotein Sm D2	T
PADG_05900	Transcription elongation factor 1 homolog	T
PADG_03406	Transcription initiation factor IIA subunit 2	T
**PROTEIN SYNTHESIS**		
PADG_00878	Isoleucine-tRNA ligase	T
PADG_08472	Lysine--tRNA ligase	T
PADG_02484	Valine-tRNA ligase	T
PADG_03326	40S ribosomal protein S9	T
PADG_04315	40S ribosomal protein S24	2.05
PADG_07583	40S ribosomal protein S21	T
PADG_01914	60S ribosomal protein L35	T
PADG_04065	60S ribosomal protein L36	T
PADG_07803	60S ribosomal protein L12	T
PADG_02056	Ribosomal protein L7/L12	1.80
PADG_03856	Ribosomal protein L15	1.43
PADG_04075	rRNA processing protein RRP9	1.70
PADG_01160	Eukaryotic translation initiation factor 3 subunit M	T
PADG_06110	Translation initiation factor SUI1	1.40
PADG_05404	Deoxyhypusine hydroxylase	T
PADG_01565	Calnexin	T
**PROTEIN FATE**		
PADG_03967	Proteasome component C	T
PADG_03982	Proteasome endopeptidase complex	T
PADG_06290	Proteasome subunit alpha type	T
PADG_08095	26S proteasome regulatory subunit rpn-8	6.36
PADG_02637	Biquitin-conjugating enzyme	T
PADG_07953	Peptidyl-prolyl cis-trans isomerase	T
**PROTEIN WITH BINDING FUNCTION OR COFACTOR REQUIREMENT**		
PADG_01032	DNA-binding protein, 42 kDa	T
**UNCLASSIFIED PROTEIN**		
PADG_11833	Hypothetical protein	T
PADG_06593	Hypothetical protein	T
PADG_11352	Hypothetical protein	T

PADG_11111	Hypothetical protein	T
PADG_11424	Hypothetical protein	T
PADG_00828	Hypothetical protein	T
PADG_00951	Hypothetical protein	T
PADG_00060	Hypothetical protein	1.60
PADG_00092	Hypothetical protein	T
PADG_00688	Hypothetical protein	T
PADG_00694	Hypothetical protein	2.92
PADG_02181	Hypothetical protein	T
PADG_02338	Hypothetical protein	1.51
PADG_01363	Hypothetical protein	T
PADG_01473	Hypothetical protein	1.48
PADG_01503	Hypothetical protein	T
PADG_08402	Hypothetical protein	T
PADG_02847	Hypothetical protein	T
PADG_02981	Hypothetical protein	T
PADG_03095	Hypothetical protein	3.25
PADG_03104	Hypothetical protein	1.32
PADG_03176	Hypothetical protein	T
PADG_03526	Hypothetical protein	1.72
PADG_04165	Hypothetical protein	T
PADG_04193	Hypothetical protein	T
PADG_04907	Hypothetical protein	T
PADG_04994	Hypothetical protein	T
PADG_05167	Hypothetical protein	T
PADG_06136	Hypothetical protein	4.35
PADG_02764	Hypothetical protein	T
PADG_06196	Hypothetical protein	T
PADG_06997	Hypothetical protein	T
PADG_07506	Hypothetical protein	1.80
PADG_07515	Hypothetical protein	1.51
PADG_07670	Hypothetical protein	T
PADG_07682	Hypothetical protein	T
PADG_07870	Hypothetical protein	T
PADG_07896	Hypothetical protein	T
PADG_03149	Hypothetical protein	T

* T: Proteins identified only in the treatment condition (cells cultured in the presence of AOS).

Table S2. Proteins downregulated by *P. brasiliensis* after exposure to AOS

Acess Number	Biological process	Fold Change
**METABOLISM**		
**Metabolism aminoacid**		

PADG_00685	3-hydroxy-3-methylglutaryl coenzyme A synthase	C
PADG_03167	3-isopropylmalate dehydratase	C
PADG_08468	4-hydroxyphenylpyruvate dioxygenase	0.50
PADG_08328	5-methyltetrahydropteroyltriglutamate-homocysteine S-methyltransferase	0.72
PADG_00888	Argininosuccinate synthase	0.59
PADG_08262	Asparagine synthase	C
PADG_04570	Branched-chain-amino-acid aminotransferase 3	0.68
PADG_03984	Glutamine-fructose-6-phosphate transaminase	0.25
PADG_03825	NAD-specific glutamate dehydrogenase	0.56
PADG_01615	Homocitrate synthase. mitochondrial	C
PADG_07366	Methylcrotonoyl-CoA carboxylase subunit alpha	C
PADG_06144	Saccharopine dehydrogenase	0.58
PADG_01928	S-adenosylmethionine synthase	0.59
PADG_03522	S-methyl-5'-thioadenosine phosphorylase	C
PADG_05301	Cystathionine beta-synthase	0.61
**Lipid, fatty acid and isoprenoid metabolism**		
PADG_08651	Peroxisomal hydratase-dehydrogenase-epimerase	C
PADG_00254	Fatty acid synthase subunit alpha	C
PADG_00255	FAD dependent oxidoreductase superfamily	0.67
PADG_05783	Farnesyl pyrophosphate synthetase	0.64
PADG_00608	Formyl-coenzyme A transferase	0.75
**C-compound and carbohydrate metabolism**		
PADG_04900	Trehalose-6-phosphate synthase	C
PADG_08474	Mannose-1-phosphate guanyltransferase subunit beta-A	0.18
PADG_01993	Acetoacetate-CoA ligase	C
**Nitrogen, sulfur and selenium metabolism**		
PADG_07674	Carbonic anhydrase	C
PADG_07010	Urease accessory protein UreG	0.51
PADG_00446	Oxidoreductase 2-nitropropane dioxygenase family	C
**Metabolism of vitamins, cofactors, and prosthetic groups**		
PADG_00443	Dihydropteroate synthase	0.63
**Nucleotide/nucleoside/nucleobase metabolism**		
PADG_05321	Mitochondrial nuclease	0.02
**Secondary metabolism**		
PADG_04603	Spermidine synthase	C
PADG_08034	Dienelactone hydrolase family protein	C
**ENERGY**		

**Tricarboxylic-acid pathway**		
PADG_02805	Isocitrate dehydrogenase mitochondrial	0.59
PADG_04993	Citrate synthase subunit 1	0.64
PADG_11845	Aconitate hydratase mitochondrial	0.45
PADG_02260	Succinate-CoA ligase subunit alpha mitochondrial	C
**Electron transport and membrane-associated energy conservation**		
PADG_08391	Plasma membrane ATPase	C
PADG_04729	ATP synthase subunit d mitochondrial	C
PADG_05402	ATP synthase Mitochondrial F1F0	C
PADG_05436	Cytochrome b-c1 complex subunit Rieske. mitochondrial	C
PADG_04397	Cytochrome c oxidase subunit 4. mitochondrial	0.76
**Glycolysis and gluconeogenesis**		
PADG_03118	Glucose-6-phosphate 1-epimerase	0.73
PADG_01896	Phosphoglycerate kinase	0.54
PADG_00780	Ribose-phosphate pyrophosphokinase	0.61
**CELL RESCUE. DEFENSE AND VIRULENCE**		
PADG_00324	Catalase	C
PADG_01711	Hsp90 co-chaperone AHA1	0.42
PADG_07715	Hsp90-like protein	0.30
PADG_00765	Hsp98-like protein	C
PADG_03963	30 kDa heat shock protein	0.63
PADG_04034	Chaperone DnaJ	0.45
PADG_05628	Protein disulfide-isomerase domain	0.49
PADG_05122	Prenylcysteine oxidase	C
**BIOGENESIS OF CELLULAR COMPONENTS**		
PADG_00128	Tubulin alpha chain	0.39
PADG_08413	Tubulin alpha chain	C
PADG_12077	Actin	0.46
PADG_12076	Actin	0.51
PADG_05538	Actin	0.70
PADG_00344	Actin cytoskeleton protein	0.71
**CELL CYCLE AND DNA PROCESSING**		
PADG_00849	Nuclear segregation protein Bfr1	0.76
**CELL FATE**		
PADG_06763	Septum formation protein Maf	0.38
PADG_06336	Cell lysis protein cwl1	0.52
PADG_07726	Periodic tryptophan protein	C
**CELLULAR COMMUNICATION/SIGNAL TRANSDUCTION MECHANISM**		
PADG_02017	Calmodulin	C
PADG_08342	GTP-binding protein ypt1	C
PADG_08337	GTP-binding protein rhoA	C

PADG_00554	GTP-binding protein rho2	C
PADG_04810	GTP-binding nuclear protein	0.53
PADG_05608	GTP-binding protein ypt7	0.68
**CELLULAR TRANSPORT, TRANSPORT FACILITIES AND TRANSPORT ROUTES**		
PADG_01440	ADP.ATP carrier protein	C
PADG_00622	ATPase GET3	0.30
PADG_04100	Clathrin heavy chain	C
PADG_02022	Clathrin light chain	C
PADG_02134	Coatomer subunit epsilon	C
PADG_07508	Phosphatidylinositol transporter	0.64
PADG_01243	Rab GDP dissociation inhibitor	C
PADG_01567	Sorting nexin 3	C
PADG_05898	Rheb small monomeric GTPase RhbA	0.31
**PROTEIN SYNTHESIS**		
PADG_06525	40S ribosomal protein S1	0.66
PADG_00335	40S ribosomal protein S14	0.70
PADG_02445	40S ribosomal protein S15	0.70
PADG_12324	40S ribosomal protein S19	0.70
PADG_06680	40S ribosomal protein S22	0.55
PADG_06048	40S ribosomal protein S27	0.73
PADG_08605	40S ribosomal protein S28	0.42
PADG_04030	60S acidic ribosomal protein P0	0.45
PADG_08244	60S acidic ribosomal protein P1	0.51
PADG_02446	60S acidic ribosomal protein P2	0.63
PADG_02249	60S ribosomal protein L2	0.68
PADG_12253	60S ribosomal protein L3	0.09
PADG_11379	60S ribosomal protein L5	0.74
PADG_04848	60S ribosomal protein L8-B	C
PADG_03778	60S ribosomal protein L10-A	0.73
PADG_11227	60S ribosomal protein L13	0.76
PADG_05338	60S ribosomal protein L18-B	0.76
PADG_03873	60S ribosomal protein L20	C
PADG_04588	Ribosomal protein L22	0.53
PADG_04449	60S ribosomal protein L23	0.73
PADG_05883	60S ribosomal protein L25	0.73
PADG_05939	60S ribosomal protein L27a	0.64
PADG_01083	60S ribosomal protein L32	0.75
PADG_02828	Ribosomal protein	C
PADG_05721	Ribosomal protein L4	0.65
PADG_00785	Ribosomal protein S15	C
PADG_05388	Asparagine-tRNA ligase	C
PADG_04962	Aspartate-tRNA(Asn) ligase	0.61
PADG_00002	Alanine-tRNA ligase	0.70
PADG_03689	Tyrosine-tRNA ligase	C

PADG_05848	Glycine-tRNA ligase	C
PADG_00821	Glutamate-tRNA ligase	C
PADG_03440	Proline-tRNA ligase	0.05
PADG_01558	Histidyl-tRNA synthetase	0.68
PADG_01406	Phenylalanine-tRNA ligase. beta subunit	0.64
PADG_00692	Elongation factor 1-alpha	0.64
PADG_08125	Elongation factor 2	0.61
PADG_07229	Elongation factor 3 partial mRNA	C
PADG_01949	Elongation factor Tu	0.55
PADG_07977	Eukaryotic translation initiation factor 1A. Y-chromosomal	C
PADG_04083	Eukaryotic translation initiation factor 2 subunit gamma	C
PADG_04016	Eukaryotic translation initiation factor 3 subunit A	C
PADG_02296	Eukaryotic translation initiation factor 3 subunit F	C
PADG_04251	Eukaryotic translation initiation factor 3 subunit I	C
PADG_04057	Eukaryotic translation initiation factor 3 subunit J	0.57
PADG_00342	Eukaryotic translation initiation factor 3 subunit L	C
PADG_00932	Eukaryotic translation initiation factor eIF3	C
PADG_07888	Eukaryotic translation initiation factor 5A	C
PADG_04210	Translation initiation factor 2 subunit beta	0.65
PADG_11711	ATP-dependent RNA helicase eIF4A	0.30
PADG_05787	ATP-dependent RNA helicase FAL1	C
PADG_04672	ATP-dependent RNA helicase SUB2	C
**PROTEIN FATE**		
PADG_04234	26S proteasome non-ATPase regulatory subunit 8	C
PADG_00599	26S protease regulatory subunit 6A	C
PADG_06851	26S proteasome non-ATPase regulatory subunit 11	0.23
PADG_12469	26S proteasome non-ATPase regulatory subunit 6	C
PADG_03680	Proteasome subunit beta type	C
PADG_02833	ADP-ribosylation factor	C
PADG_08048	T-complex protein 1 subunit beta	C
PADG_00050	T-complex protein 1 subunit delta	0.45
PADG_08484	T-complex protein 1 subunit epsilon	C
PADG_00602	Arginine N-methyltransferase	0.68
PADG_06359	Bleomycin hydrolase	C

PADG_05837	E3 ubiquitin ligase complex SCF subunit sconC	0.07
PADG_04092	Peptidyl-prolyl cis-trans isomerase	C
**UNCLASSIFIED PROTEIN**		
PADG_03147	Hypothetical protein	C
PADG_12408	Hypothetical protein	C
PADG_11705	Hypothetical protein	C
PADG_11793	Hypothetical protein	C
PADG_11832	Hypothetical protein	0.70
PADG_11128	Hypothetical protein	C
PADG_11413	Hypothetical protein	0.64
PADG_00855	Hypothetical protein	C
PADG_00222	Hypothetical protein	C
PADG_00449	Hypothetical protein	C
PADG_00528	Hypothetical protein	C
PADG_00541	Hypothetical protein	C
PADG_02092	Hypothetical protein	C
PADG_02206	Hypothetical protein	0.54
PADG_02352	Hypothetical protein	C
PADG_01488	Hypothetical protein	0.47
PADG_01702	Hypothetical protein	C
PADG_01935	Hypothetical protein	C
PADG_02048	Hypothetical protein	C
PADG_03431	Hypothetical protein	0.66
PADG_02456	Hypothetical protein	C
PADG_02854	Hypothetical protein	C
PADG_02878	Hypothetical protein	C
PADG_02967	Hypothetical protein	0.59
PADG_03115	Hypothetical protein	C
PADG_03170	Hypothetical protein	C
PADG_03210	Hypothetical protein	0.69
PADG_03219	Hypothetical protein	C
PADG_03244	Hypothetical protein	C
PADG_03325	Hypothetical protein	0.75
PADG_03609	Hypothetical protein	C
PADG_03703	Hypothetical protein	C
PADG_03999	Hypothetical protein	C
PADG_04307	Hypothetical protein	C
PADG_04311	Hypothetical protein	0.63
PADG_06033	Hypothetical protein	0.70
PADG_04869	Hypothetical protein	C
PADG_05034	Hypothetical protein	0.44
PADG_04657	Hypothetical protein	0.73
PADG_04730	Hypothetical protein	C
PADG_05227	Hypothetical protein	C

PADG_05341	Hypothetical protein	C
PADG_05598	Hypothetical protein	0.63
PADG_05750	Hypothetical protein	0.76
PADG_05893	Hypothetical protein	0.74
PADG_06117	Hypothetical protein	C
PADG_06305	Hypothetical protein	C
PADG_06888	Hypothetical protein	0.36
PADG_06969	Hypothetical protein	C
PADG_07714	Hypothetical protein	0.64
PADG_08715	Hypothetical protein	0.74
PADG_07264	Hypothetical protein	C
PADG_00824	Hypothetical protein	0.68

* C: Proteins identified only in the control condition (cells cultured in the absence of AOS).
